# Supplementary material for: Histone dynamics mediate DNA unwrapping and sliding in nucleosomes
Source: Nat Commun. 2021 Apr 22;12:2387. doi: 10.1038/s41467-021-22636-9 (PMC8062685; doi:10.1038/s41467-021-22636-9)
Supplement: Supplementary file 1 — Supplementary Information [file 41467_2021_22636_MOESM1_ESM.pdf]

SUPPLEMENTARY INFORMATION

Histone dynamics mediate DNA unwrapping and sliding in nucleosomes

Grigoriy A. Armeev[1,2], Anastasia S. Kniazeva[2], Galina A. Komarova[3], Mikhail P. Kirpichnikov[1,4], and Alexey K. Shaytan[1,2,5,*]

[1]Department of Biology, Lomonosov Moscow State University, 1-12 Leninskie Gory, Moscow, 119991, Russia
[2]Sirius University of Science and Technology, 1 Olympic Ave, Sochi, 354340, Russia
[3]Department of Physics, Lomonosov Moscow State University, 1-2 Leninskie Gory, Moscow, 119991, Russia
[4]Shemyakin-Ovchinnikov Institute of Bioorganic Chemistry, Russian Academy of Sciences, 16/10 Miklukho-Maklaya st., Moscow, 117997, Russia
[5]Bioinformatics Lab, Faculty of Computer Science, HSE University, 11 Pokrovsky boulvar, Moscow, 109028, Russia

These authors contributed equally: Grigoriy A. Armeev, Anastasia S. Kniazeva.
*email: shaytan_ak@mail.bio.msu.ru

Supplementary Note 1: Detailed simulated systems' description.

NCP$_{147}$ - based on an X-ray structure of NCP with PDB ID 1KX5 [1]. It has 147 bp DNA derived from a human alpha-satellite DNA sequence repeat. Octamer is based on canonical histones of *X. laevis.* DNA sequence forms two symmetric palindromic 73 bp segments in the structure plus one base pair is strictly at the dyad. The system has full-length histone tails. Symmetry related histone tails were put in identical starting conformations.

NCP$_{147}^{tt}$ - analogous to NCP$_{147}$, but histone tails are truncated in this system.

NCP$_{146}^{tt}$ - based on an X-ray structure of NCP with PDB ID 1AOI [2]. It has 146 bp of palindromic DNA derived from human X-chromosome alpha-satellite DNA repeat. Octamer is based on canonical histones of *X. laevis.* Histone tails are truncated in this system.

NCP$_{145}^{tt}$ - based on an X-ray structure of NCP with PDB ID 3LZ0 [3]. It has 145 bp of Widom 601 strong positioning DNA sequence. Octamer is based on canonical histones of *X. laevis.* Histone tails are truncated in this system.

NCP$_{147}^{fixed}$ - same as NCP$_{147}$, but histone folds' C-α-atoms are fixed (restraints of 1000 kJ mol^{-1} nm^{-}2).

System parameters are further presented in Supplementary Table 1.

Supplementary Table 1: Parameters of the simulated systems.

Name	PDB ID[a]	Box volume, nm^3	Atom number	Water molecules	Ions number, Na/Cl	Ionic strength by water volume, mM	Ionic strength by box volume, mM	Bulk ionic strength,[b] mM	Minimum periodic image distance, nm	Time, μs
NCP_{147}	1KX5	2658	267K	81K	363/219	150	136	172	2.089	15
NCP^{tt}_{147}	1KX5	2914	293K	90K	468/242	149	137	168	2.53	10
NCP^{tt}_{145}	3LZ0	2817	283K	87K	459/237	151	139	168	2.47	15
NCP^{tt}_{146}	1AOI	2641	265K	81K	445/221	151	138	178	3.52	8
NCP^{fixed}_{147}	1KX5	2425	245K	73K	343/199	151	136	170	1.48	8

[a] PDB database ID used to derive the simulated system model.
[b] Calculated at a distance beyond 1 nm from the nucleosome.
[c] See Figure 1e for the location of truncation sites.

Supplementary Table 2: Trajectory averaged number of contacts between DNA and histone globular core or histone tails for NCP_{147} system

**Classification method**	**Contact types**	**Histone core**[a]	**Histone tails**
All contacts		601	1219
Nucleotide parts	Phosphate	458	583
	Base	10	288
	Sugar	133	347
Amino acids parts	Backbone	200	466
	Side chain	401	753
Interaction types	Hydrophobic	22	105
	Salt bridges	34	29
	Polar	66	152
	hydrogen bonds	34	263
DNA groove	Major	1	123
	Minor	9	162

[a] Core residues are non-tail residues. See tail regions definition in Figure 1e.

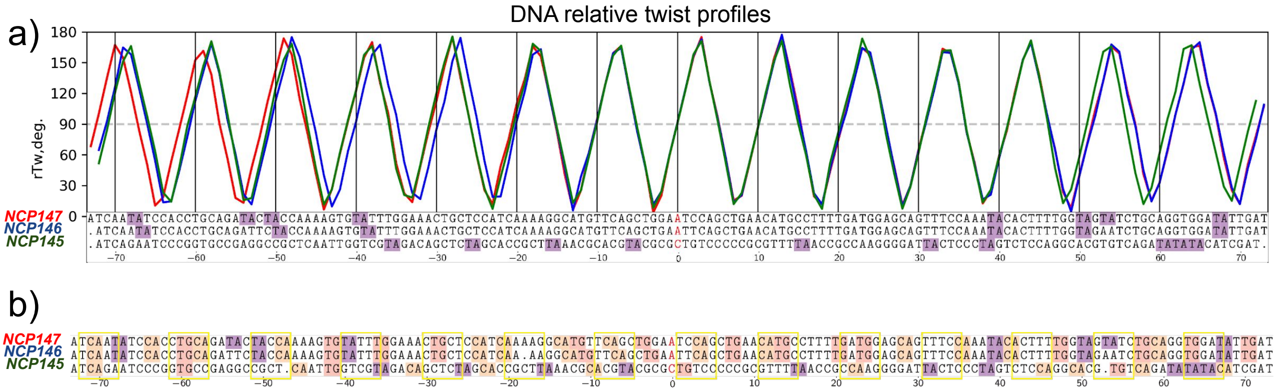

Supplementary Figure 1: a) Sequence alignment (by the dyad position) of the DNA sequences (top chain) used in the simulated systems and their relative twist profiles (rTw) in X-ray structures (indicating the orientation of DNA base pairs relative to the octamer surface - see Methods). Red nucleotides mark the dyad position, flexible TA dinucleotides are highlighted in purple; b) Structure based sequence alignment for the top DNA strand (chain I) generated by USCF Chimera [4]. Indels around SHL ±5 are detected (the results for the bottom strand are nearly equivalent). Yellow frames mark regions, where a minor groove faces towards the histone octamer; pyrimidine/purine dinucleotides are highlighted (TA - purple, CA - apricot, TG - pink).

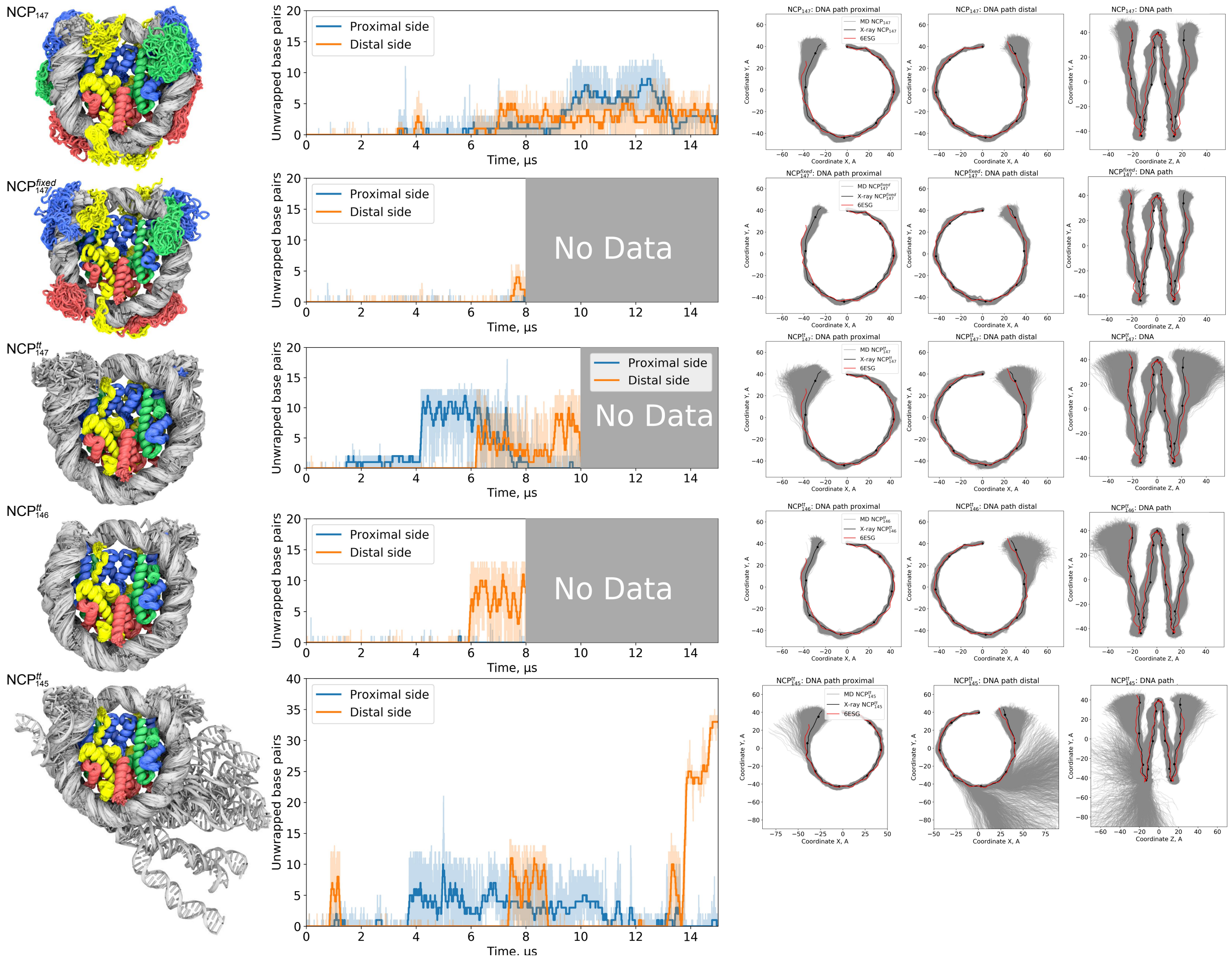

Supplementary Figure 2: Overview of the dynamics of the simulated systems. First column: overlay of snapshots along the whole MD trajectory for each system (every 100 ns). Second column: plots of the extent of DNA unwrapping (as estimated by displacement of base pair centers). Right three columns: 2D projections of DNA path (base pair centers) in nucleosome reference frame for frames along the MD trajectory.

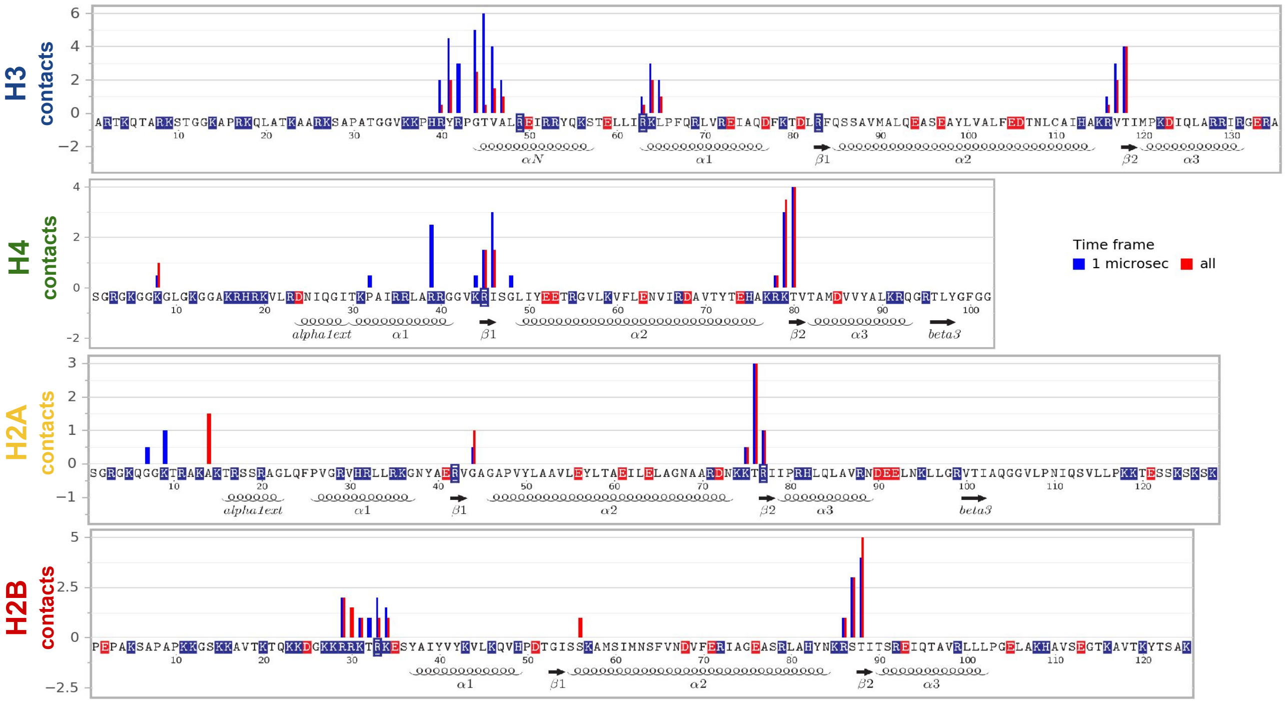

Supplementary Figure 3: Number of stable atom-atom contacts between histone residues and nucleosomal DNA plotted along the histone sequences for NCP_{147} simulation. Stable atom-atom contacts are defined as those present in at least 40% of MD frames (the threshold is lower than for stable residue-nucleotide contacts because atom-atom contacts are less persistent). Contacts are averaged over symmetry-related histone chains. The data is shown for the first 1 μs and the full 15 μs trajectory.

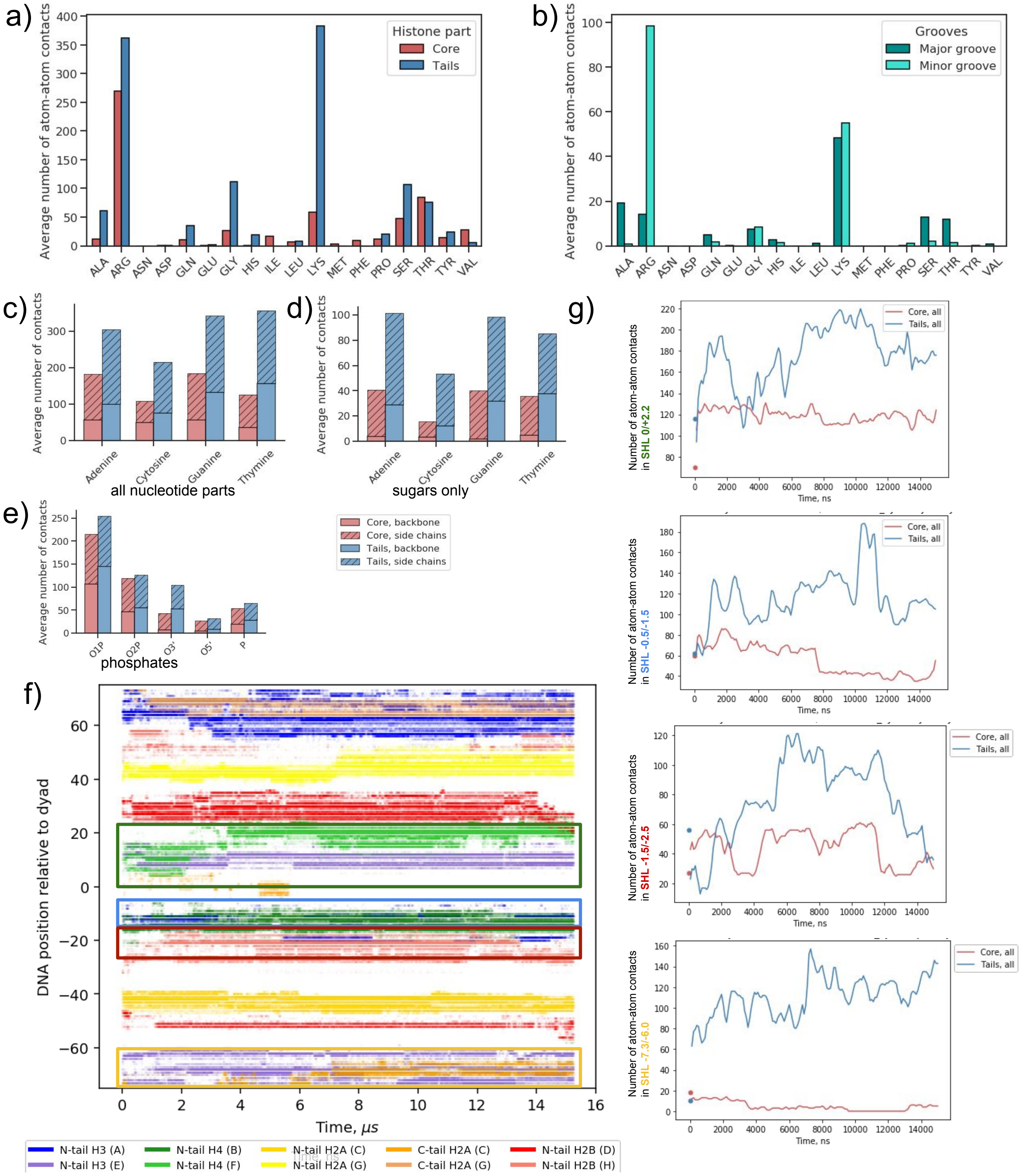

Supplementary Figure 4: Evolution and statistics of histone-DNA atom-atom contacts for NCP_{147} simulations. a) Number of contacts classified by histone part and amino acid residue; b) contacts between histones and DNA bases classified by amino acid residue and DNA major/minor groove; c) contacts between histones and DNA classified by nucleotide; d) contacts between histones and DNA sugar moieties classified by nucleotide; e) contacts between histones and DNA phosphates classified by phosphate atoms; f) a map representing the evolution of interactions between histone tails and different positions in nucleosomal DNA; g) evolution of atom-atom contacts between core and tail parts of histones for different segments of nucleosomal DNA defined by their SHLs and highlighted by color boxes in panel f.

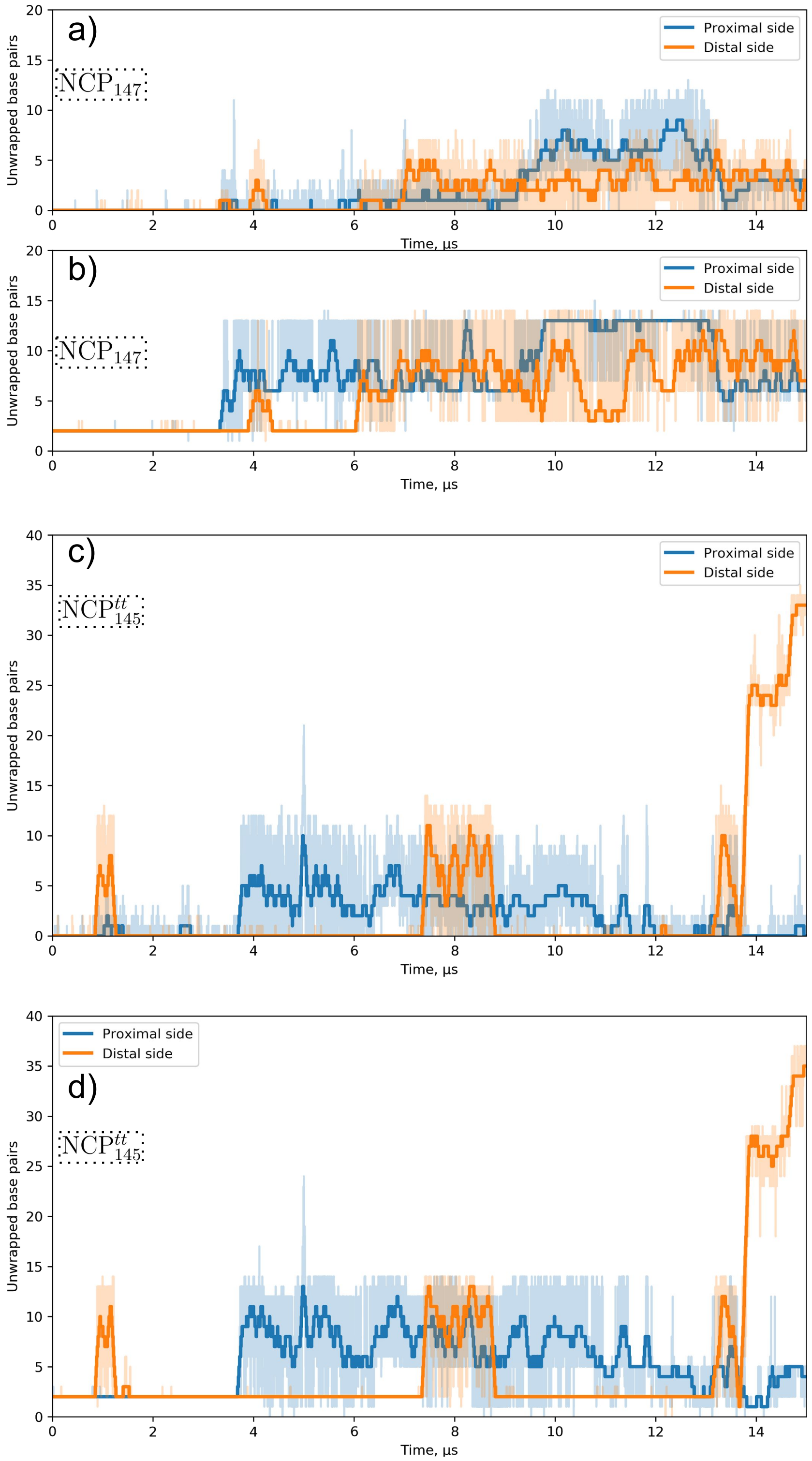

Supplementary Figure 5: Comparison of two approaches to quantify DNA unwrapping for NCP$_{147}$ and NCP$^{tt}_{145}$ systems. a),c) distance criterion: displacement of all the base pairs of the segment by more than $7\mathring{A}$ from the DNA path in the corresponding X-ray structure. b),d) contacts criterion: loss of contacts with the globular core (non-tail part) of the histone octamer.

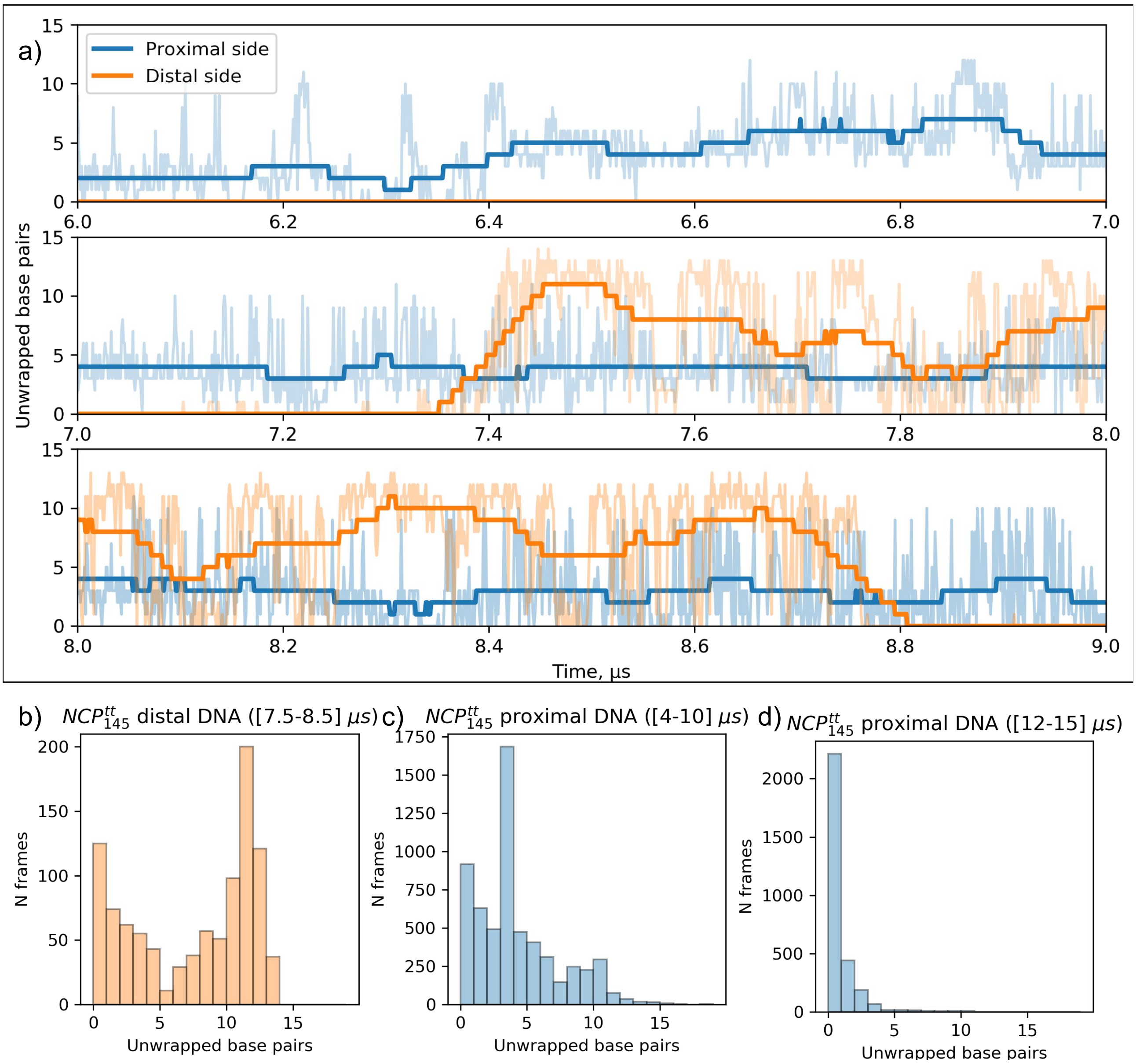

Supplementary Figure 6: A detailed view of DNA unwrapping dynamics for NCP^{tt}_{145} simulation. a) A zoom-up view of DNA unwrapping dynamics between 6-9 μs. b) Histogram of different unwrap values for the distal DNA end sampled during period 7.5-8.5 μs. c) Histogram of different unwrap values for the proximal DNA end sampled during period 4-10 μs. d) Histogram of different unwrap values for the proximal DNA end sampled during period 12-15 μs.

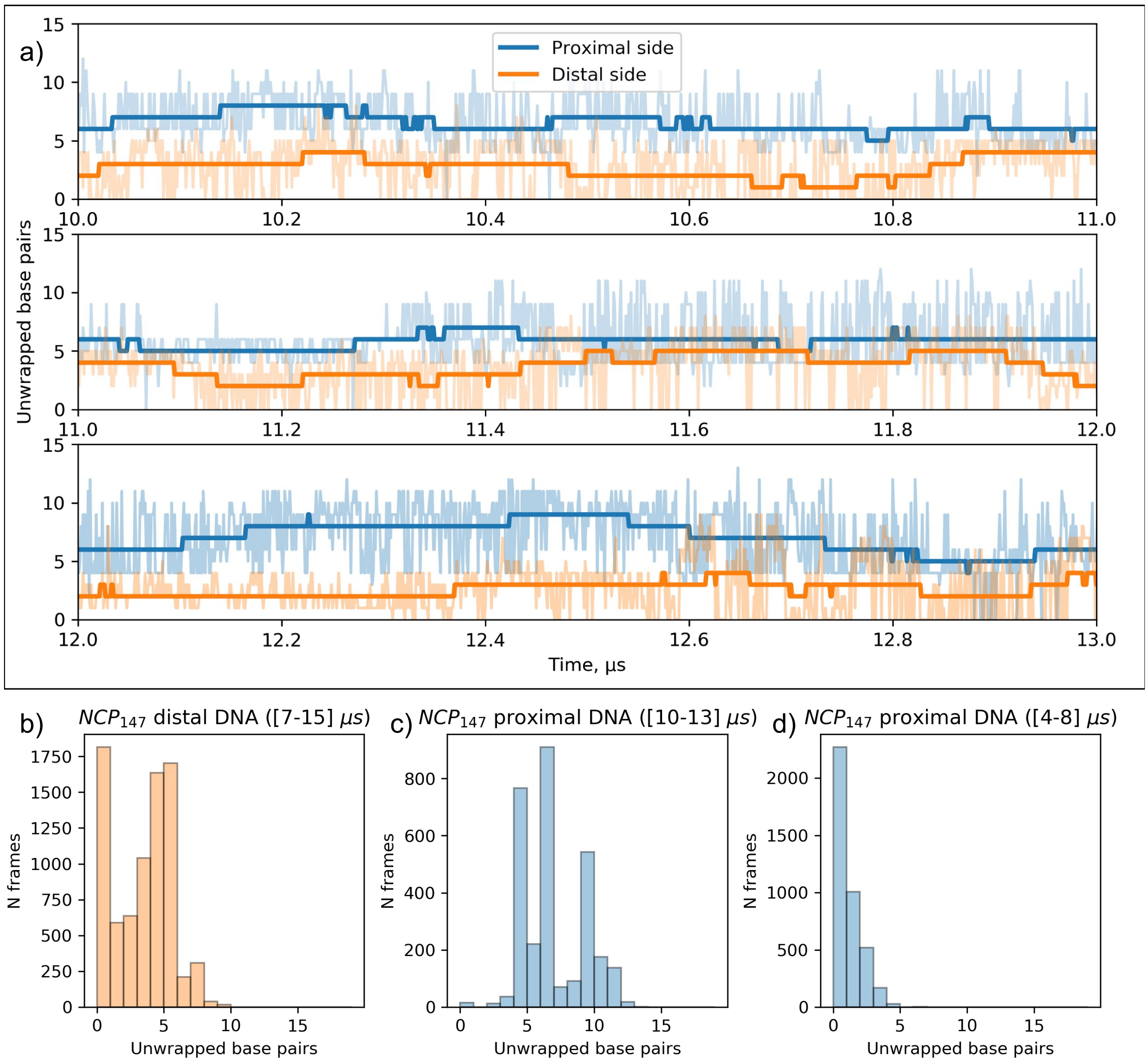

Supplementary Figure 7: A detailed view of DNA unwrapping dynamics for NCP_{147} simulation. a) A zoom-up view of DNA unwrapping dynamics between 10-13 μs. b) Histogram of different unwrap values for the distal DNA end sampled during period 7-15 μs. c) Histogram of different unwrap values for the proximal DNA end sampled during period 10-13 μs. d) Histogram of different unwrap values for the proximal DNA end sampled during period 4-8 μs.

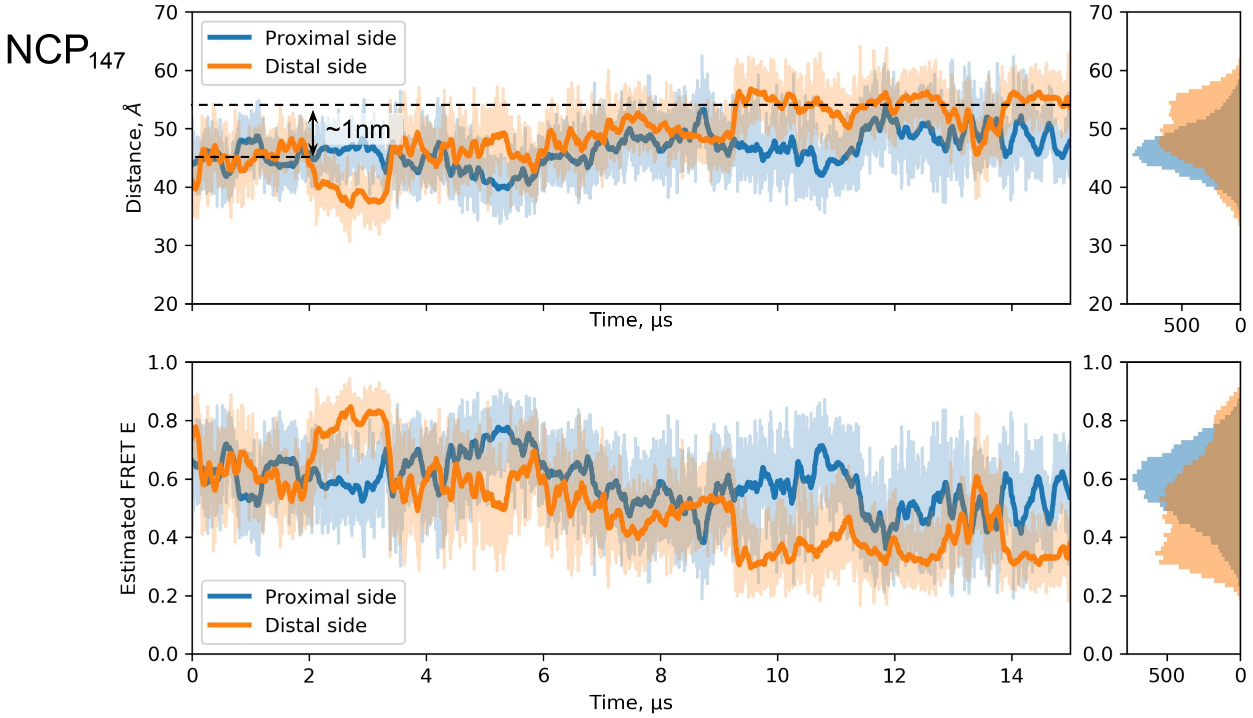

Supplementary Figure 8: Distances (top panel) and simulated FRET efficiencies (bottom panel) between FRET labels placed on DNA positions -73 and 2 for the proximal side and -2 and 73 for the distal side following ref. [5] for NCP$_{147}$. We estimated the distances between the fluorescent dyes' attachment sites as the distances between O5′ atoms of the sugar-phosphate backbone of the respective nucleotides. FRET efficiencies were calculated as $E = \dfrac{1}{1 + (r/R_0)^6}$ were r is distance between dyes and Förster radius $R_0 = 49\mathring{A}$ as in ref. [5].

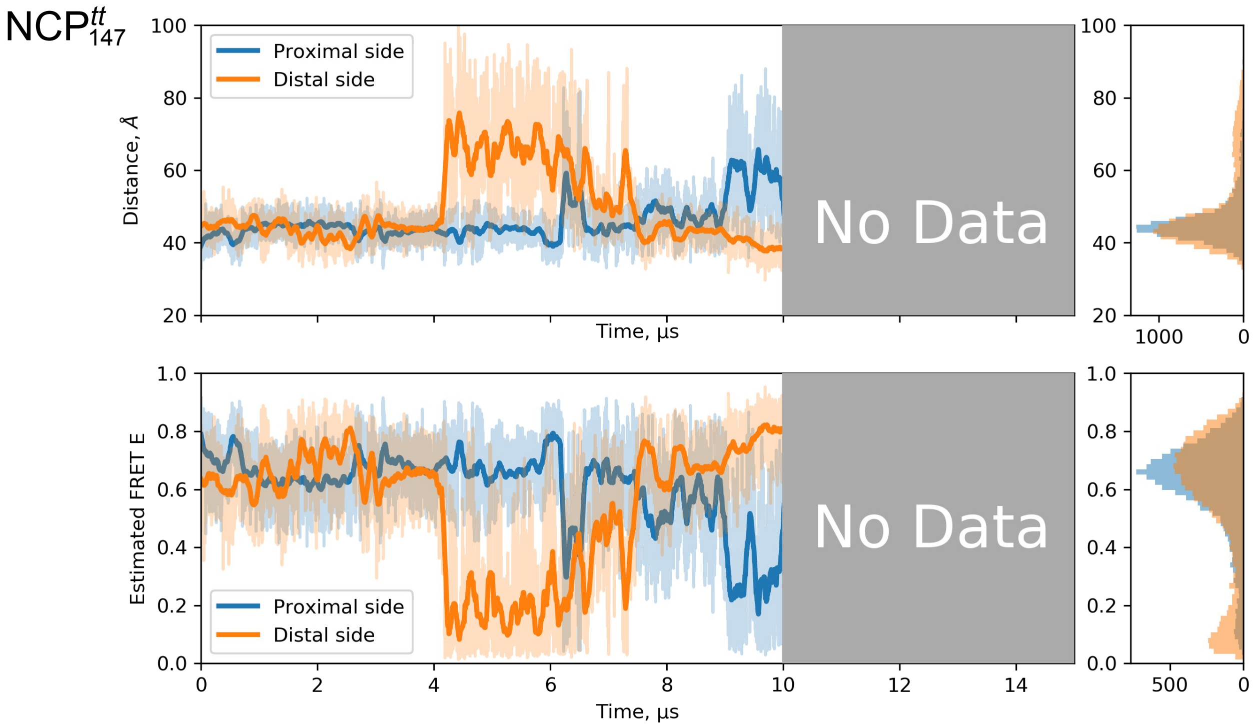

Supplementary Figure 9: Distances (top panel) and simulated FRET efficiencies (bottom panel) between FRET labels placed on DNA positions -73 and 2 for proximal side and -2 and 73 for distal side following ref. [5] for NCP$^{tt}_{147}$. Distances and FRET efficiencies are measured as in 8.

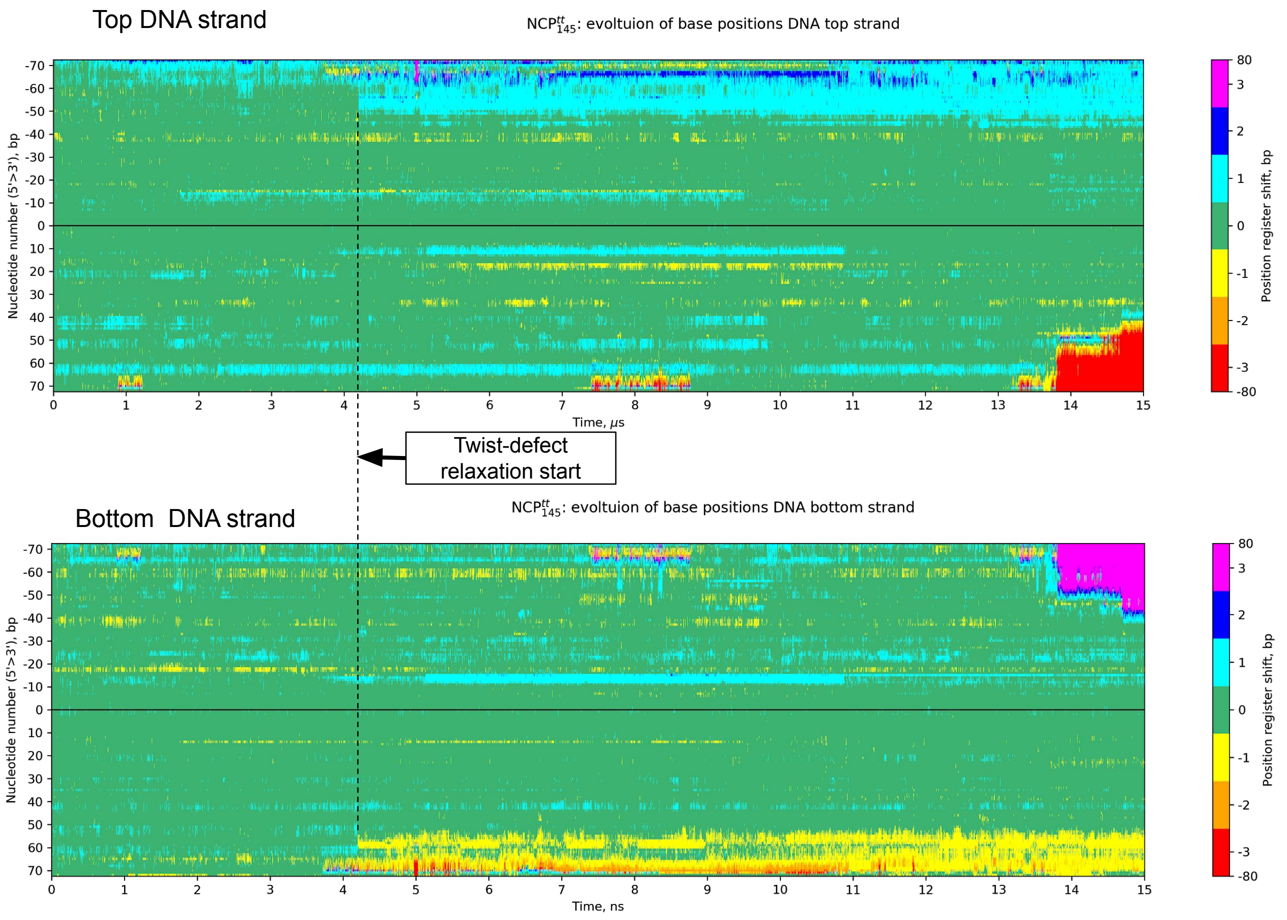

Supplementary Figure 10: DNA strand position register shift during simulations of NCP$^{tt}_{145}$. The plot is similar to Figure 7b, but shows data for the full range of the bottom and top DNA strands.

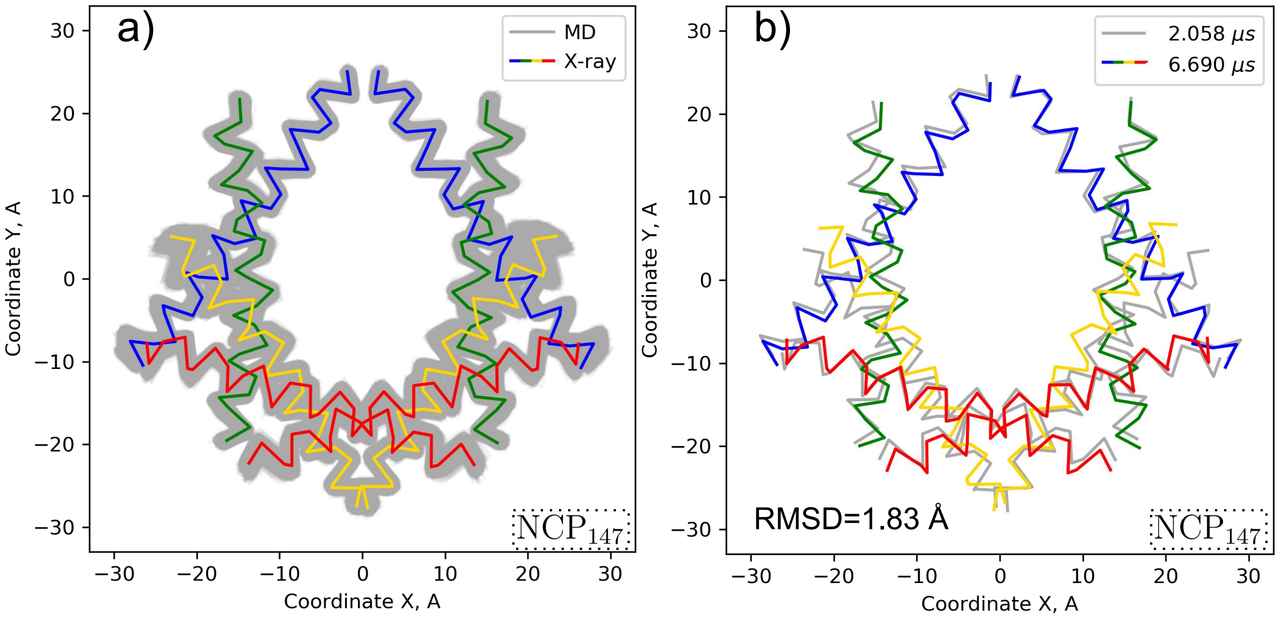

Supplementary Figure 11: Analysis of histone octamer plasticity in NCP$_{147}$ simulation. The description is similar to Figure 8

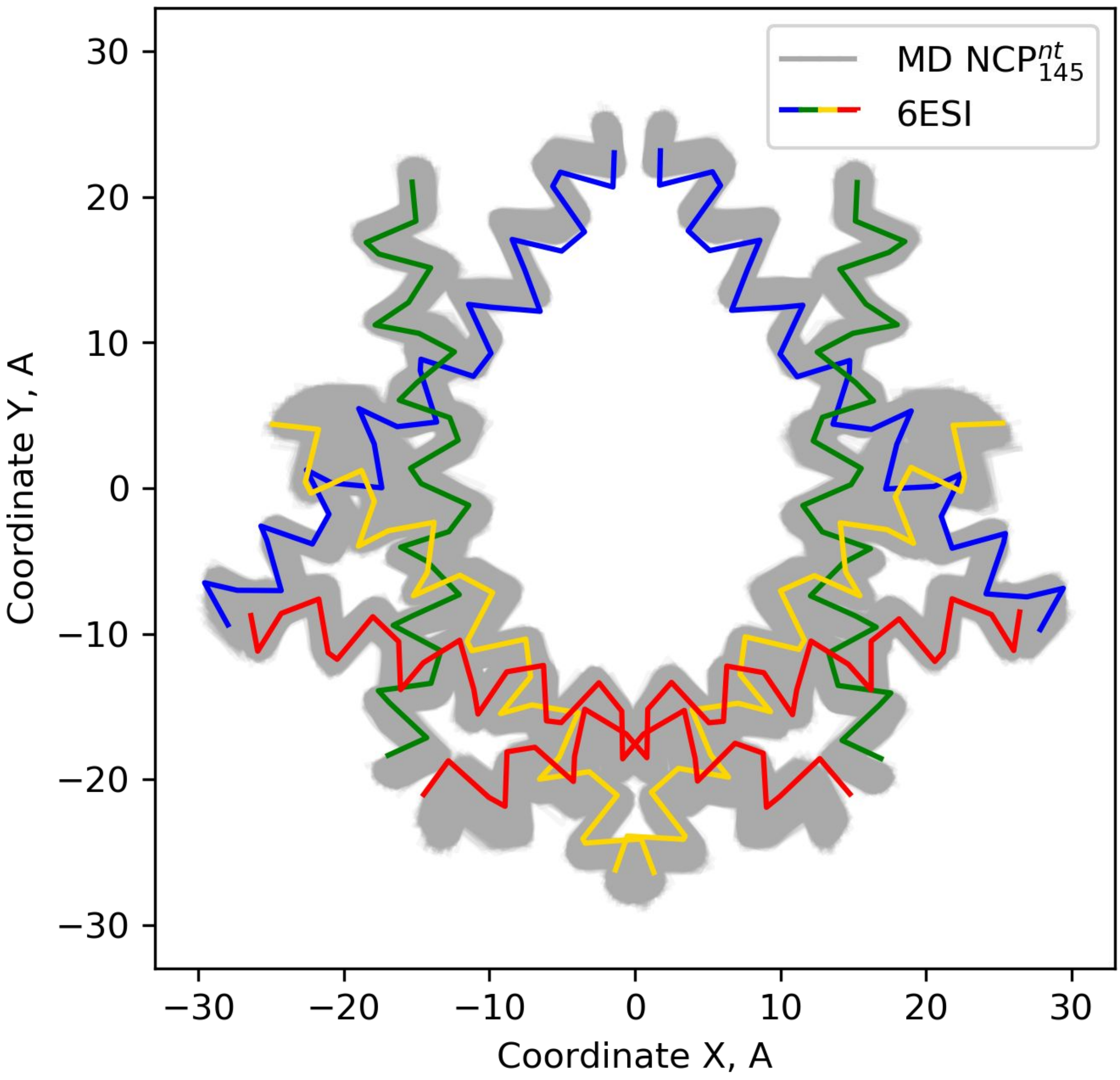

Supplementary Figure 12: 2D projections of the histone α2-helices from NCP$^{tt}_{145}$ simulation vs. their projections in the recently reported deformed (squeezed by 8% along the dyad) NCP structure seen in cryo-EM (PDB ID 6FQ6).

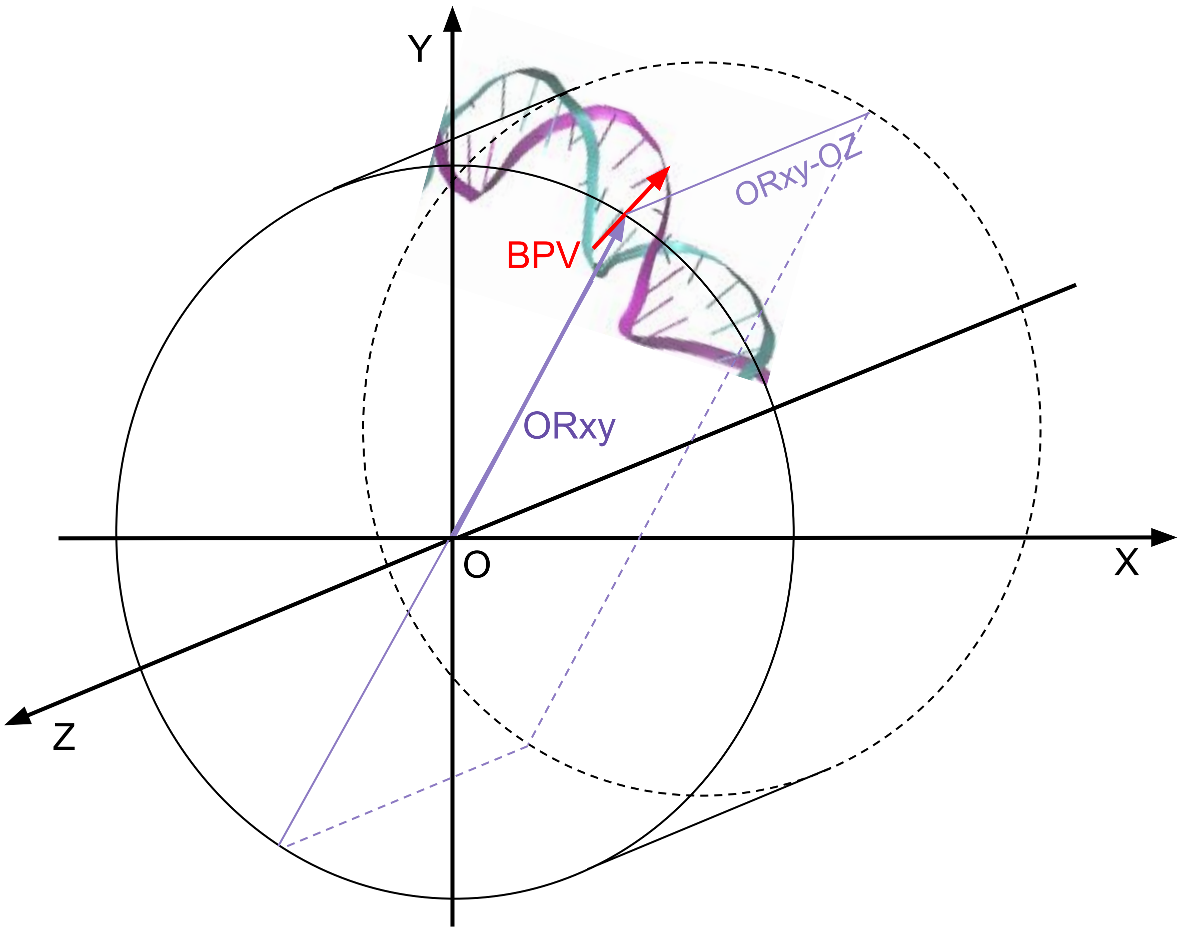

Supplementary Figure 13: Schematic description of relative twist (rTw) determination. **BPV** - base pair vector, **ORxy** - radial vector in cylindrical coordinate system, **OZ** - superhelical axis. rTw is defined as the angle between **BPV** projection onto the ORxy-OZ plane and OXY plane.

Supplementary References

[1] Davey, C. A., Sargent, D. F., Luger, K., Maeder, A. W. & Richmond, T. J. Solvent mediated interactions in the structure of the nucleosome core particle at 1.9 a resolution **319**, 1097–1113 (2002).

[2] Luger, K., Mäder, A. W., Richmond, R. K., Sargent, D. F. & Richmond, T. J. Crystal structure of the nucleosome core particle at 2.8 A resolution **389**, 251–260 (1997).

[3] Vasudevan, D., Chua, E. Y. D. & Davey, C. A. Crystal Structures of Nucleosome Core Particles Containing the '601' Strong Positioning Sequence **403**, 1–10 (2010).

[4] Pettersen, E. F. *et al.* UCSF Chimera–a visualization system for exploratory research and analysis **25**, 1605–1612 (2004).

[5] Wei, S., Falk, S. J., Black, B. E. & Lee, T.-H. A novel hybrid single molecule approach reveals spontaneous DNA motion in the nucleosome **43**, e111 (2015).
