# Supplementary material for: Histone dynamics mediate DNA unwrapping and sliding in nucleosomes
Source: Nat Commun. 2021 Apr 22;12:2387. doi: 10.1038/s41467-021-22636-9 (PMC8062685; doi:10.1038/s41467-021-22636-9)
Supplement: Supplementary file 3 — Description of Additional Supplementary Files [file 41467_2021_22636_MOESM3_ESM.docx]

**Description of Additional Supplementary Files**

File Name: Supplementary Movie 1

Description: Dynamics of NCP_147_ system

File Name: Supplementary Movie 2

Description: Dynamics of NCP^tt^_147_ system

File Name: Supplementary Movie 3

Description: Dynamics of NCP^tt^_145_ system

File Name: Supplementary Movie 4

Description: Dynamics of NCP^tt^_146_ system

File Name: Supplementary Movie 5

Description: Dynamics of NCP^fixed^_147_ system

File Name: Supplementary Movie 6

Description: Dynamics of contacts between anchor residues and nucleosomal DNA in NCP_147_ system.

File Name: Supplementary Movie 7

Description: Dynamics of contacts between anchor residues and nucleosomal DNA in NCP^tt^_147_ system.

File Name: Supplementary Movie 8

Description: Dynamics of contacts between anchor residues and nucleosomal DNA in NCP^tt^_145_ system.

File Name: Supplementary Movie 9

Description: Dynamics of contacts between anchor residues and nucleosomal DNA in NCP^tt^_146_ system.

File Name: Supplementary Movie 10

Description: Dynamics of contacts between anchor residues and nucleosomal DNA in NCP^fixed^_147_ system.
