# Supplementary material for: Histone dynamics mediate DNA unwrapping and sliding in nucleosomes
Source: Nat Commun. 2021 Apr 22;12:2387. doi: 10.1038/s41467-021-22636-9 (PMC8062685; doi:10.1038/s41467-021-22636-9)
Supplement: Supplementary file 14 — Reporting Summary [file 41467_2021_22636_MOESM14_ESM.pdf]

nature research

Corresponding author(s): Alexey K. Shaytan

Last updated by author(s): Mar 12, 2021

# Reporting Summary

Nature Research wishes to improve the reproducibility of the work that we publish. This form provides structure for consistency and transparency in reporting. For further information on Nature Research policies, see our Editorial Policies and the Editorial Policy Checklist.

## Statistics

For all statistical analyses, confirm that the following items are present in the figure legend, table legend, main text, or Methods section.

| n/a | Confirmed | |
|---|---|---|
| ☒ | ☐ | The exact sample size ($n$) for each experimental group/condition, given as a discrete number and unit of measurement |
| ☒ | ☐ | A statement on whether measurements were taken from distinct samples or whether the same sample was measured repeatedly |
| ☒ | ☐ | The statistical test(s) used AND whether they are one- or two-sided<br>*Only common tests should be described solely by name; describe more complex techniques in the Methods section.* |
| ☒ | ☐ | A description of all covariates tested |
| ☒ | ☐ | A description of any assumptions or corrections, such as tests of normality and adjustment for multiple comparisons |
| ☒ | ☐ | A full description of the statistical parameters including central tendency (e.g. means) or other basic estimates (e.g. regression coefficient) AND variation (e.g. standard deviation) or associated estimates of uncertainty (e.g. confidence intervals) |
| ☒ | ☐ | For null hypothesis testing, the test statistic (e.g. $F$, $t$, $r$) with confidence intervals, effect sizes, degrees of freedom and $P$ value noted<br>*Give P values as exact values whenever suitable.* |
| ☒ | ☐ | For Bayesian analysis, information on the choice of priors and Markov chain Monte Carlo settings |
| ☒ | ☐ | For hierarchical and complex designs, identification of the appropriate level for tests and full reporting of outcomes |
| ☒ | ☐ | Estimates of effect sizes (e.g. Cohen's $d$, Pearson's $r$), indicating how they were calculated |

*Our web collection on statistics for biologists contains articles on many of the points above.*

## Software and code

Policy information about availability of computer code

| | |
|---|---|
| Data collection | GROMACS 2018.1 was used for molecular dynamics simulations, available at http://www.gromacs.org |
| Data analysis | Python 3 with following key libraries: MDAnalysis v 0.20.0, pandas 1.0.1, numpy 1.17.3.<br>VMD version 1.9.3 available at http://www.ks.uiuc.edu/Research/vmd/<br>3DNA version v2.4.2-2019feb26 available at http://x3dna.org/<br>Python scripts implementing the analysis available at https://intbio.github.io/Armeev_et_al_2021 or https://doi.org/10.5281/zenodo.4590559 |

For manuscripts utilizing custom algorithms or software that are central to the research but not yet described in published literature, software must be made available to editors and reviewers. We strongly encourage code deposition in a community repository (e.g. GitHub). See the Nature Research guidelines for submitting code & software for further information.

## Data

Policy information about availability of data

All manuscripts must include a data availability statement. This statement should provide the following information, where applicable:

- Accession codes, unique identifiers, or web links for publicly available datasets
- A list of figures that have associated raw data
- A description of any restrictions on data availability

Data supporting the findings of this manuscript are available from the corresponding author upon reasonable request. A reporting summary for this Article is available as a Supplementary Information file. Trajectories and protocols are available for preview and download from GitHub at https://intbio.github.io/Armeev_et_al_2021 and are archived via Zenodo at https://doi.org/10.5281/zenodo.4590559.

# Field-specific reporting

Please select the one below that is the best fit for your research. If you are not sure, read the appropriate sections before making your selection.

☒ Life sciences ☐ Behavioural & social sciences ☐ Ecological, evolutionary & environmental sciences

For a reference copy of the document with all sections, see [nature.com/documents/nr-reporting-summary-flat.pdf](nature.com/documents/nr-reporting-summary-flat.pdf)

# Life sciences study design

All studies must disclose on these points even when the disclosure is negative.

| | |
|---|---|
| Sample size | We have performed molecular dynamics simulations for 5 systems. These systems vary slightly with respect to the DNA sequence, presence/ absence of histone tails. The key finding and statements of the paper are compatible with the dynamical behavior observed in all simulated systems. |
| Data exclusions | No data was excluded |
| Replication | Our simulation require large amounts of computational time at the supercomputer. Instead of replicating each simulation verbatim we opted for longer simulations of each system and simulating several variations of the system to be confident that our result do not depend on the slight variation in the system composition. |
| Randomization | Randomization is not relevant to our study since it did not include collection of any statistical samples. All the simulations data was directly available for the analysis. |
| Blinding | Blinding is not relevant to our study since it did not include collection of any statistical samples. All the simulations data was directly available for the analysis. |

# Reporting for specific materials, systems and methods

We require information from authors about some types of materials, experimental systems and methods used in many studies. Here, indicate whether each material, system or method listed is relevant to your study. If you are not sure if a list item applies to your research, read the appropriate section before selecting a response.

## Materials & experimental systems

| n/a | Involved in the study |
|---|---|
| ☒ | ☐ Antibodies |
| ☒ | ☐ Eukaryotic cell lines |
| ☒ | ☐ Palaeontology and archaeology |
| ☒ | ☐ Animals and other organisms |
| ☒ | ☐ Human research participants |
| ☒ | ☐ Clinical data |
| ☒ | ☐ Dual use research of concern |

## Methods

| n/a | Involved in the study |
|---|---|
| ☒ | ☐ ChIP-seq |
| ☒ | ☐ Flow cytometry |
| ☒ | ☐ MRI-based neuroimaging |



April 2020
